# Relationships between Degree of Polymerization and Antioxidant Activities: A Study on Proanthocyanidins from the Leaves of a Medicinal Mangrove Plant *Ceriops tagal*

**DOI:** 10.1371/journal.pone.0107606

**Published:** 2014-10-14

**Authors:** Hai-Chao Zhou, Nora Fung-yee Tam, Yi-Ming Lin, Zhen-Hua Ding, Wei-Ming Chai, Shu-Dong Wei

**Affiliations:** 1 Key Laboratory of the Ministry of Education for Coastal and Wetland Ecosystems, Xiamen University, Xiamen, China; 2 Department of Biology and Chemistry, City University of Hong Kong, Hong Kong SAR, China; 3 Futian-CityU Mangrove R&D Centre, City University of Hong Kong Shenzhen Research Institute, Shenzhen, China; Università della Calabria, Italy

## Abstract

Tannins from the leaves of a medicinal mangrove plant, *Ceriops tagal*, were purified and fractionated on Sephadex LH-20 columns. ^13^C nuclear magnetic resonance (^13^C-NMR), reversed/normal high performance liquid chromatography electrospray ionization mass spectrometry (HPLC-ESI MS) and matrix-assisted laser desorption/ionization time-of-flight mass spectrometry (MALDT-TOF MS) analysis showed that the tannins were predominantly B-type procyanidins with minor A-type linkages, galloyl and glucosyl substitutions, and a degree of polymerization (DP) up to 33. Thirteen subfractions of the procyanidins were successfully obtained by a modified fractionation method, and their antioxidant activities were investigated using 2,2-diphenyl-1-picrylhydrazyl (DPPH) scavenging capacity and ferric reducing antioxidant power (FRAP) method. All these subfractions exhibited potent antioxidant activities, and eleven of them showed significantly different mean DP (mDP) ranging from 1.43±0.04 to 31.77±1.15. Regression analysis demonstrated that antioxidant activities were positively correlative with mDP when around mDP <10, while dropped and then remained at a level similar to mDP = 5 with around 95 µg ml^−1^ for DPPH scavenging activity and 4 mmol AAE g^−1^ for FRAP value.

## Introduction

*Ceriops tagal*, a mangrove plant species in the Family of Rhizophoraceae, is one of the medicinal plants in East and Southeast Asia and is often used as a tradition herb to treat diseases such as hemorrhages and malignant ulcers [1], [2]. The phytochemistry of *C. tagal* has been reported, however, the work mainly focused on the isolation and identification of terpenoid compounds in root, with little attention on leaf tannins [3], [4]. The high level of tannins in the leaves of Rhizophoraceae is known to deter feeding by herbivores [5], but leaf tannins also show a diversity of other biological activities [6]. The unexplored tannins could be novel potential resources of bioactive compounds in mangrove plants. So far, the chemical properties of *C. tagal* tannins have not yet been determined and the structure-activity relationships of tannins are still not clear.

Tannins comprised as much as 20–40% dry weight in the leaf and bark of mangrove plants [7], [8]. Compared with hydrolysable tannins, condensed tannins (proanthocyanidins) are more abundant. They are commonly found in mangrove plants [9], [10], [11], [12], [13], [14] and are also the main component of the polyphenols in our diet [15]. Because of their antioxidant activities and other potentially health-promoting qualities, proanthocyanidins have attracted more and more research interests in recent years [16], [17], [18], [19]. Proanthocyanidins are oligomers and polymers of flavan-3-ol that are bound together with B-type and A-type linkages [15]. The chemistry and biological features of proanthocyanidins largely depend on their structure, particularly the molecular weight that is also expressed as degree of polymerization (DP) [20], [21]. Ariga et al. [22] found that the ability to scavenge free radicals was proportional to DP for simple flavonoid oligomers. Hagerman et al. [23] proved that tannins with highly polymerized and many hydroxyl groups are more potent antioxidants than the simple phenolics. Recently, some findings reported that the increasing DP may enhance the antioxidant power of condensed tannins [24], [25]. However, those previous works either limitedly detected the simple oligomers or directly studied the bulk mixture of polymers. The relationships between DP and antioxidant activity of proanthocyanidins, therefore, remained largely unknown.

The present study, therefore, aims to (I) achieve a complete structural characterization of tannins from the leaf of a mangrove plant (*C. tagal*) by a combination of analytical techniques, including ^13^C-NMR, MALDI-TOF MS, thiolysis degradation, and reversed/normal-phase HPLC-ESI MS; and (II) establish an efficient fractionation method to obtain a series of proanthocyanidins with different DP; and (III) explore the possible relationships between DP and antioxidant activity of condensed tannins.

## Materials and Methods

### 2.1. Chemicals and Materials

Water used in this experiment was purified on a Millipore Milli-Q apparatus. HPLC grade dichloromethane, acetonitrile (CH_3_CN), methanol, trifluoroacetic acid (TFA), acetic acid, and all analytical grade solvents (acetone, methanol, n-Butanol etc.) were obtained from Sinopharm (Sinopharm, Shanghai, China). Sephadex LH-20, Folin-Ciocalteu reagents, acetone-*d_6_*, deuteroxide (D_2_O), Amberlite IRP-64 cation-exchange resin, cesium chloride (CsCl), 2,5-dihydroxybenzoic acid (DHB), benzylmercaptan, 2,2-diphenyl-1-picrylhydrazyl (DPPH), 2,4,6-tripyridyl-S-triazine (TPTZ), ascorbic acid (AA), and all HPLC standards were purchased from Sigma (St. Louis, MO, USA). The mature leaves (the third pair with fully expanded and dark green) of a mangrove plant *C. tagal* (Rhizophoraceae *Ceriops*), were collected from a mangrove forest in the Dongzhai harbor (19°56′N, 110°34′E), Hainan, China. The leaves were immediately freeze-dried, ground, and stored at −20°C prior to analysis.

### 2.2. Extraction, Purification and Fractionation of Mangrove Tannins

**Figure 1:**
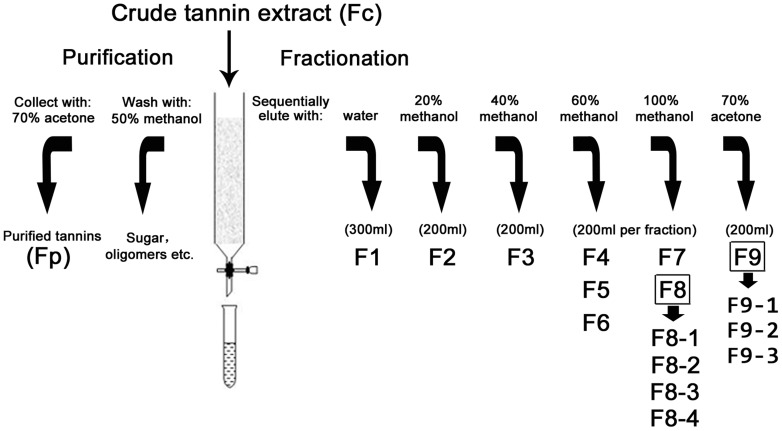
Sephadex LH-20 column chromatography for purification and fractionation of tannins from *C. tagal* leaves. Subfraction 8 (F8) and Subfraction 9 (F9) were further fractionated into F8-1 to F8-4 and F9-1 to F9-3, respectively.

The extraction procedure was conducted according to the method of Zhou et al. [25] The crude tannins extract (Fc) obtained was applied to a Sephadex LH-20 column (50×1.5 cm i.d.). The procedure of purification and fractionation of F_C_ was shown in Figure 1. Purified tannins (Fp) and nine subfractions (F1 to F9) were evaporated under vacuum to remove organic solvents followed by freeze-dried. The last two subfractions (F8 and F9) were re-loaded on a Sephadex LH-20 column (50×1.0 cm i.d.) to yield seven more subfractions as shown below.

### 2.3. Determination of Total Phenolics and Extractable Condensed Tannins

The procedure described by Zhou et al. [25] to estimate the total phenolics and extractable condensed tannins of the entire leaf of *C. tagal* was used, and the concentrations were determined by Folin-Ciocalteu [26] and butanol-HCl method [27], respectively. The content of the total phenolics (TP) and extractable condensed tannins (ECT) were calculated using the purified tannins (Fp) obtained from the purification of crude tannins extract (Fc) as the standard. Both of them were expressed as mg Fp equivalents per gram dry weight of leaves.

### 2.4. ^13^C-NMR Analysis

The obtained purified tannins from *C. tagal* leaves were dissolved in acetone-*d_6_*/D_2_O. A Varian Metcury-600 spectrometer (Palo Alto, CA, USA) was employed in the 150 MHz mode [10].

### 2.5. Reversed-phase HPLC-ESI MS Analysis Followed by Thiolysis

The modified method described by Zhou et al. [9] was carried out to characterize Fp and obtain the mDP for each subfraction. Condensed tannins were thiolysis degraded by benzylmercaptan, and then the degradation products were analyzed on an Agilent 1200 system (Agilent, Palo Alto, CA, USA) interfaced to a QTRAP 3200 (Applied Biosystems, Foster, USA) with a 250 mm×4.6 mm i.d. 5.0 µm Hypersil ODS column (Elite, Dalian, China).

### 2.6. Normal-phase HPLC-ESI MS Analysis

Normal-phase HPLC-ESI-MS analysis was conducted according to the method of Hellstrom et al. [28] with minor modifications. The HPLC-MS equipment consisted of an Agilent 1200 liquid chromatograph system as described above with a 250 mm×4.6 mm i.d. 5.0-µm Silica Luna column (Phenomenex, Darmstadt, Germany).

### 2.7. MALDI-TOF MS Analysis

The MALDI-TOF MS analyses were performed on a Bruker Reflex III instrument (Germany). The measurements were carried out using the conditions reported in the previous work [29]. Reflectron modes coupled with linear modes were further applied to show the different DP distribution of condensed tannins.

### 2.8. Determination of Antioxidant Activities

Antioxidant activities of each subfraction were evaluated by DPPH radical scavenging activity (DPPH method) [30] and ferric ion reducing antioxidant power (FRAP method) [31]. Their results were expressed in EC_50_ value (µg mL^−1^) and mmol ascorbic acid equivalents per gram of each subfraction (mmol AAE g^−1^), respectively. Procedures were modified according the method of Zhou et al. [32].

### 2.9. Statistical Analysis

Results of antioxidant activity were expressed as mean ± standard deviation of three independent determinations. A parametric one-way analysis of variance (ANOVA) was used to test any significant difference among subfractions at *P*<0.05, and all the data without transformation fulfilled the two assumptions, normal distribution and homogeneity in variance of the parametric test. All statistical analyses were performed with SPSS 17.0 for Windows.

### 2.10. Ethics Statement

Field permit issued by The Mangroves of Dongzhai Gang National Reserve in Dongzhai harbor, Hainan, China, allowed us to collect the mature leaves of *C. tagal* in this site. They also confirmed that our study did not involve any endangered or protected species.

## Results and Discussion

### 3.1. Contents of Total Phenolics and Extractable Condensed Tannins

The total phenolics and extractable condensed tannins content of *C. tagal* leaves was 27.4±2.8% and 20.1±2.0%, respectively. Phenolic compounds (including proanthocyanidins) were considered to be the major contributor to the antioxidant activity of vegetables, fruits or medicinal plants [25], [33], [34]. The high level of total phenolics and extractable condensed tannins in *C. tagal* leaves strongly suggested that some of their pharmacological effects could be attributed to the presence of these valuable constituents.

### 3.2. Structural Characterization of Purified Tannins (Fp)

**Figure 2:**
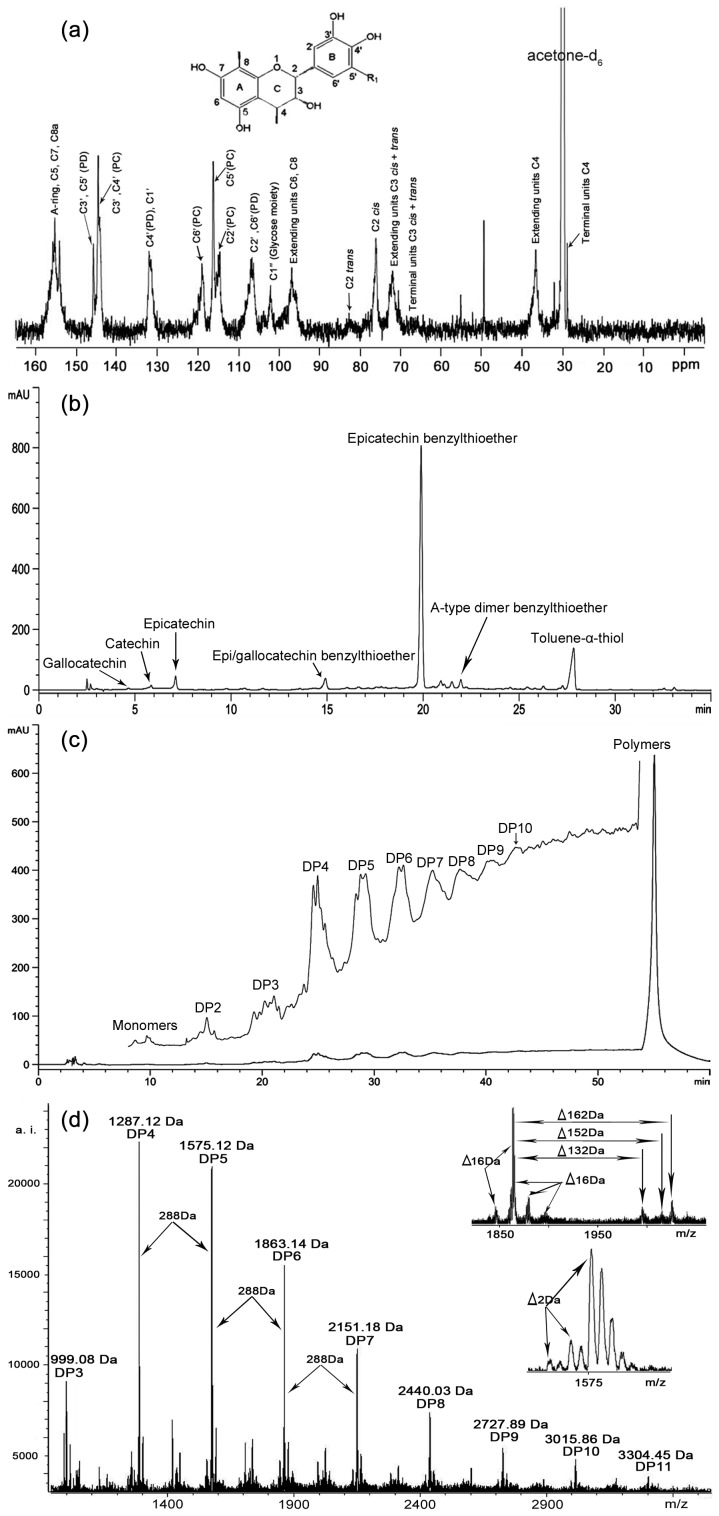
Structural characterization of purified tannins (Fp) from *C. tagal* leaves. Analyzed by (a) ^13^C-NMR, (b) reversed-phase HPLC-ESI-MS, (c) normal-phase HPLC-ESI-MS and (d) MALDI-TOF MS.

Fp from *C. tagal* leaves were initially analyzed by ^13^C-NMR spectroscopy, and the signal assignment was performed according to the method of Czochanska et al. [35] The ^13^C-NMR spectrum (Figure 2a) showed that the characteristic ^13^C peaks are consistent with that of condensed tannins with dominant procyanidin units and a minor amount of prodelphinidins. The signals near 145 ppm, which arise from quaternary C3′ and C5′ in prodelphinidins (PD), and C3′ and C4′ in procyanidin (PC) units were used to estimate the PD∶PC ratio [35]. In this experiment, direct integration of the signals at 146.2 ppm (PD units) and 145.2 ppm (PC units) gave a PD∶PC ratio of about 8∶92. The signals at 115.1 ppm (C2′), 116.1 ppm (C5′) and 119.0 ppm (C6′) further supported the presence of PC, so does signals at 107–108 ppm (C2′ and C6′) and 133.0 ppm (C4′) for PD. The structural diversity of the linkage (A type and B type) and the stereoisomer of catechin and epicatechin units were apparent from the spectrum. Specially, C5, C7, and C8a carbons of procyanidins appeared at 150 to 160 ppm [36]. The region between 70 and 90 ppm was sensitive to the stereochemistry of the C-ring. The ratio of the 2,3-*cis* to 2,3-*trans* isomers could be determined through the distinct differences in their respective C2 chemical shifts. C2 gave a resonance at 76.7 ppm for the *cis* form and 83.6 ppm for the *trans* form. From the peak areas, it was estimated that the *cis* isomer is dominant. The C3 generally have their chemical shift 66–68 ppm in terminal units, and 72–74 in extending units [37]. Theoretically, the intensity of the C3 signal in terminal units relative to that of the signal in extension units could be used for elucidating the polymer chain length [35]. However, the application of this technique for the quantification of molecular weight suffered from inaccuracy due to the low signal-to-noise ratio [24]. Similarly, the spectra obtained in the present study also showed low signal-to-noise ratio (Figure 2a). The C4 atoms of the extension units showed a broad peak at around 37 ppm, while the terminal C4 exhibits 29 ppm [37], next to the solvent peak acetone-*d_6_*. The signal at 103.1 ppm would attribute to C1″ of the glycose moiety connected to the C3 position, while the signals of C2″ (74.0 ppm), C3″ (76.3 ppm), C4″ (71.4 ppm) and C5″ (75.7 ppm) were overlapped with the chemical shifts 70–80 ppm referred above [38]. These results thus showed that the mangrove condensed tannins of *C. tagal* leaves were predominantly constituted of epicatechin, the main constitutive monomer with some glycosides.

Although the ^13^C-NMR spectrum revealed complex structural characteristics of the condensed tannins of *C. tagal* leaves, this analytical technique hardly allows the determination of the polymer chain length and the chemical constitution of individual chains. Moreover, the sequential succession of monomer units in individual chains could not be elucidated [17], [36], [39]. To overcome these problems, further characterization was continued by means of HPLC-ESI-MS and MALDI-TOF MS.

The nature of flavan-3-ol units within proanthocyanidins was analyzed by acid catalysis in the presence of benzylmercaptan as nucleophile, which had been widely used [18], [21], [36]. In the thiolysis reaction, terminal units of condensed tannins are released as free flavanoids, while external units are distinguished as benzylthioether adducts. The extension and terminal units of proanthocyanidins could be distinguished by reversed-phase HPLC analysis (Figure 2b). The dominant peak observed was epicatechin benzylthioether along with five other smaller peaks for epicatechin, epi/gallocatechin benzylthioether, A-type dimer benzylthioether, catechin and gallocatechin. These results suggested that epicatechin was the dominant flavan-3-ol unit, which occurred as both terminal and extension units in proanthocyanidins of *C. tagal* leaves.

Normal-phase HPLC analysis was able to further reflects the heterogeneity of the proanthocyanidin mixture [28]. Although the peak band became broader in higher DP and was hard to be resolved, this normal-phase HPLC chromatogram of Fp (Figure 2c) indicated the presence of proanthocyanidins with DP from monomers to decamers (DP10). The results suggested the heterogeneity of proanthocyanidins in their constituent units and the linkages between them.

Figure 2d showed the MALDI-TOF mass spectra of Fp, recorded as Cs^+^ adducts in the positive ion reflectron mode, and the enlarged spectrums demonstrated the good resolution. These results showed large structural heterogeneity of condensed tannins in the mangrove plant *C. tagal*. The mass increased from DP3 (999.08 Da) to DP11 (3304.45 Da) by 288 Da, which corresponded to one epi/catechin monomer unit. For each multiple, substructures with 16 Da, both higher and lower, could be found (Figure 2d). These masses had been identified as heteropolymers of repeating flavan-3-ol units, which demonstrated the coexistence of epi/gallocatechin and epi/afzelechin [39]. Additionally, there were three series peaks with distances of 162 Da, 152 Da, and 132 Da. The first series (162 Da) represented glucosylated heteropolyflavans containing the flavanone [39]. The second series (152 Da) was corresponding to the addition of one galloyl group at the heterocyclic C-ring [12], [24]. And the 132 Da distance may have two different interpretations according to previous works. Reed et al. [39] interpreted it as the substitutions by pentoside (132 Da), while Xiang et al. [40] explained as quasimolecular ions [M+2Cs^+^−H]^+^ that generated by simultaneous attachment of two Cs^+^ and loss of a proton. The distinct decrease of 2 Da illustrated the nature of interflavan bonds including A-type and B-type linkages. The data from MALDI-TOF MS well agreed with the results obtained by ^13^C-NMR, reversed and normal-phase HPLC-ESI-MS analyses.

### 3.3. MALDI-TOF MS Analysis of Proanthocyanidin Subfractions

**Figure 3:**
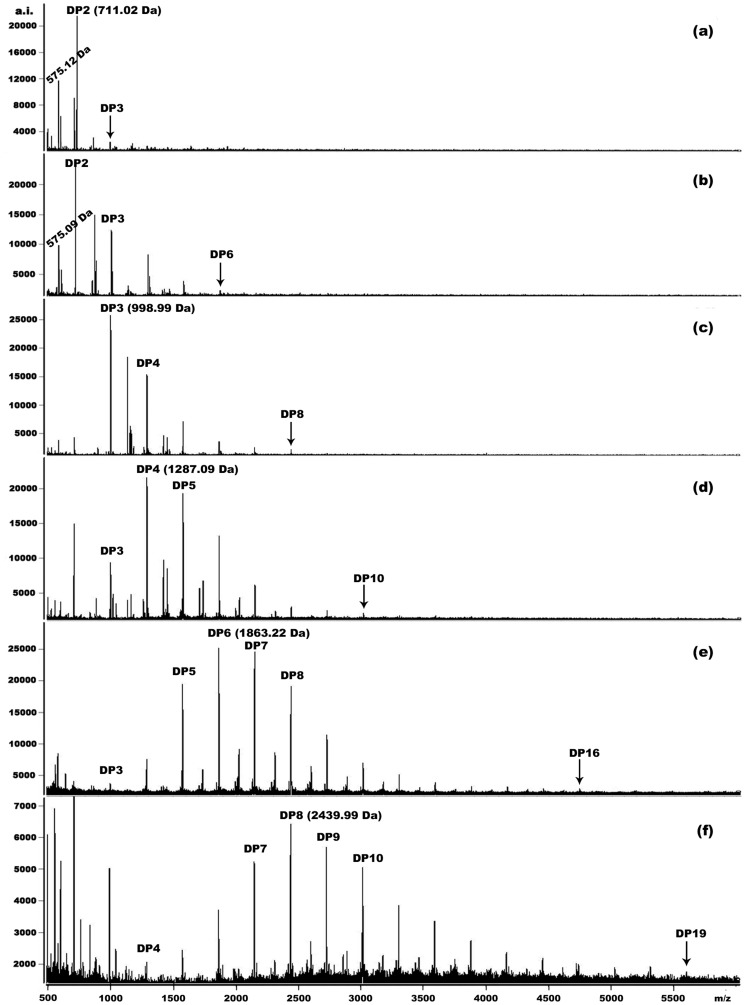
Reflectron mode MALDI-TOF mass spectra for tannin subfraction F4 (a) to F9 (f). Degree of polymerization (DP) of the predominant polymers and largest polymer in each mass spectrum of subfraction are labeled.

For the first step, ten subfractions (F1-F9) were obtained by fractionation on Sephadex LH-20 as described in methods section. After analysis by MALDI-TOF MS, fractions F1-F3 showed a cluster of unresolved peak lower than 500 Da (data not shown), which could be the sugars and other impurities. The spectra obtained by reflectron modes of the seven collected subfractions from fractions F4 (Figure 3a) to F9 (Figure 3f) clearly displayed the distinct predominant polymers and polymer range. The signals of lower oligomers were firstly detected in fraction F4 dominated by dimers (Figure 3a). Fractionation of the lower oligomers that could lead to the saturation of detector can significantly enhance the sensitivity of the detection of large polymers of proanthocyanidins under MALDI-TOF analysis [17]. Therefore, the last subfraction (F9) detected in the present study reached as high as DP19 polymer (Figure 3f), which demonstrated that the fractionation method could improve the detection of high DP polymers compared with the spectra of Fp (Figure 2d), even though it did not completely overcome the discrimination of high molecular weight polymers [17].

**Figure 4:**
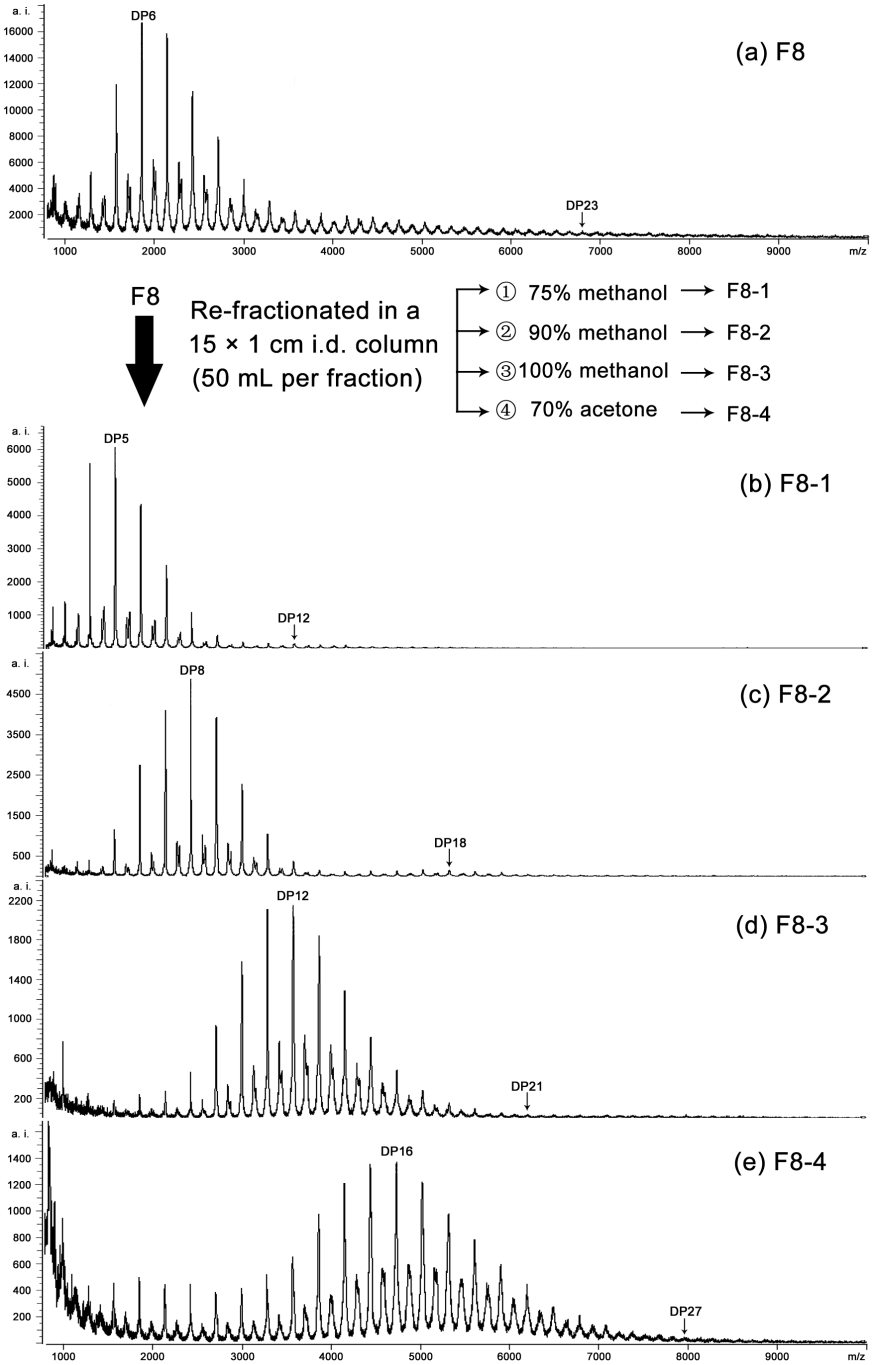
Linear mode MALDI-TOF mass spectra for tannin subfraction F8 (a). F8-1 (b) to F8-4 (e) were obtained by the further re-fractionation of F8. Degree of polymerization (DP) of the predominant polymers and largest polymer in each mass spectrum of subfraction are labeled.

**Figure 5:**
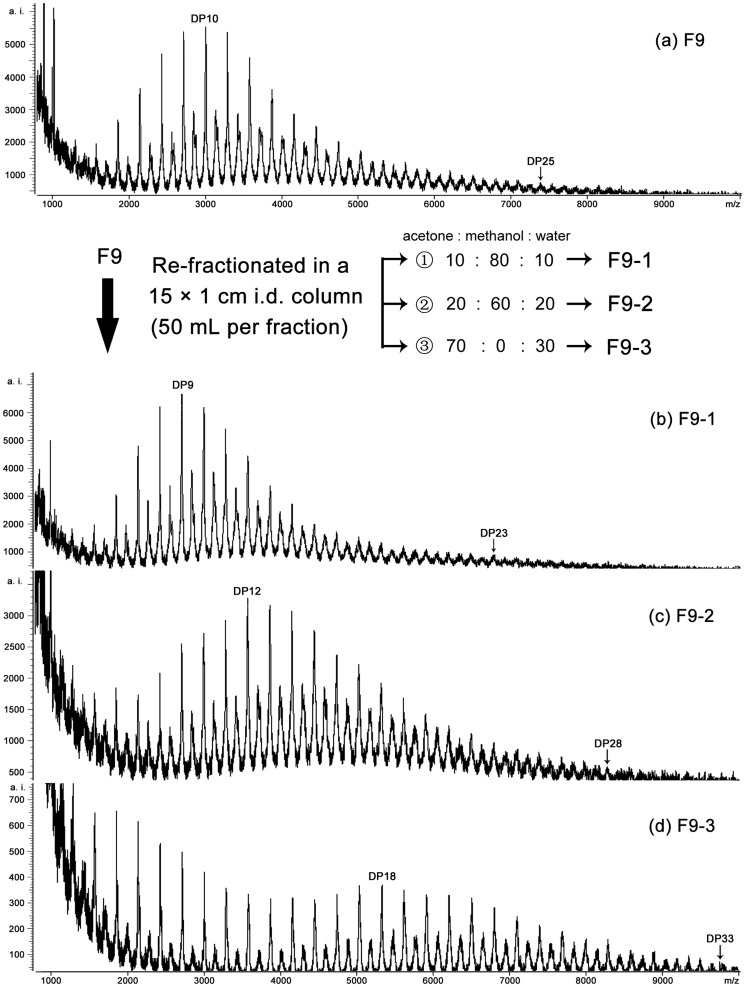
Linear mode MALDI-TOF mass spectra for tannin subfraction F9 (a). F9-1 (b) to F9-3 (d) were obtained by the further re-fractionation of F9. Degree of polymerization (DP) of the predominant polymers and largest polymer in each mass spectrum of subfraction are labeled.

Since F8 and F9 still possessed of large DP distribution (with DP3-DP16 and DP4-DP19, respectively), a refined fractionation method was conducted to re-fractionate F8 (Figure 4) and F9 (Figure 5), which yielded other four and three subfractions, respectively, as well as with increasing trends for their predominant polymers and polymer range.

After refined fractionation for F8 and F9, as high as DP27 and DP33 polymer were detected in linear spectra of F8-4 (Figure 4) and F9-3 (Figure 5), respectively. It suggested that proanthocyanidins in *C. tagal* leaves can reach DP33, or even more. The present study clearly demonstrates a higher DP and clearer DP distribution could be obtained than many previous works reporting a high DP in various plant materials using MALDI-TOF MS [17]. In the present study, however, we showed a higher DP and clearer DP distribution than those described. Both the reflectron and linear modes of MALDI-TOF MS have been successfully applied to the analysis of proanthocyanidins. The high mass resolution power of reflectron modes allows distinguishing the mass of the isotopic peaks with enough accuracy to compare with the theoretical calculated mass, while the linear mode provides better information about the mass distribution, especially for the high DP of proanthocyanidins [17], [29].

### 3.4. Mean Degree of Polymerization (mDP) Analysis of Proanthocyanidin Subfractions

Although the above MALDI-TOF mass spectra graphically showed the differences among each proanthocyanidin subfraction, it only qualitatively illustrated the changes of DP. Therefore, mDP was calculated using thiolysis degradation coupled with reversed-phase HPLC-ESI MS. Table 1 listed the mDP of 13 proanthocyanidin subfractions from *C. tagal* leaves. The mDP increased with the eluted order on Sephadex LH-20: ranging from 1.43±0.04 (F4) to 16.14±0.58 (F9), from 7.26±0.31 (F8-1) to 16.14±0.58 (F8-4) and from 14.19±0.50 (F9-1) to 31.77±1.15 (F9-3), which agreed with the results of MALDI-TOF analysis.

**Table 1:** Mean degree of polymerization (mDP) and antioxidant activities of each subfraction obtained by fractionation of proanthocyanidins from *C. tagal* leaves.

Fractions	mDP	EC_50/DPPH_ value^I^ (µg ml^−1^)	FRAP value^II^ (mmol AAE g^−1^)
F4	1.43±0.04 k	133.45±2.24 a	2.58±0.04 h
F5	2.49±0.14 j	114.74±2.24 b	3.60±0.05 g
F6	3.52±0.34 i	103.56±0.79 c	4.05±0.13 ef
F7	7.78±0.29 h	83.60±0.62 g	5.46±0.05 b
F8	10.52±0.62 g	78.13±1.14 h	5.87±0.08 a
F8-1	7.26±0.31 h	84.11±1.41 g	5.19±0.05 c
F8-2	11.72±0.56 f	97.04±1.04 d	3.83±0.07 fg
F8-3	16.84±0.82 d	86.40±0.24 fg	4.28±0.11 e
F8-4	24.62±1.15 b	98.49±1.48 d	3.80±0.01 fg
F9	16.14±0.58 d	87.14±1.68 f	4.52±0.23 d
F9-1	14.19±0.50 e	96.49±1.55 d	3.80±0.16 fg
F9-2	22.01±0.81 c	93.17±2.01 e	4.05±0.08 ef
F9-3	31.77±1.15 a	105.37±1.22 c	3.66±0.09 g

I The EC_50/DPPH_ value (µg ml^−1^) is the concentration of each subfraction at which the scavenging activity is 50%.Values with different letters in the same column are significantly different at *P*<0.05 level.

II The FRAP value (mmol AAE g^−1^) is expressed in mmol ascorbic acid equivalents per gram of each subfraction. Values with different letters in the same column are significantly different at *P*<0.05 level.

These results confirmed that the fractionation method used in this study was effective to gradually separate the plant proanthocyanidins according to DP. The occurrence of proanthocyanidins in complex mixtures makes it difficult to firmly establish the effect of molecular size (or DP) on the biologically important properties of proanthocyanidins [41]. Therefore, the distinct subfractions obtained from this fractionation method were subjected to explore the relationship between DP and antioxidant activities for proanthocyanidins.

### 3.5. Antioxidant Activities and Their Relationships with mDP

DPPH method is a reliable and reproducible method to determine the radical scavenging activity of proanthocyanidins extracted from plant or fruits [24], [42]. FRAP method is based on the redox reaction of ferric ion in the presence of a reducer. The reduction capacity of a compound may serve as a significant indicator of its potential antioxidant activity [43]. Table 1 summarized the antioxidant activities (DPPH and FRAP method) of proanthocyanidin subfractions from *C. tagal* leaves. The highest antioxidant activities were found in F8 with EC_50/DPPH_ value 78.13±1.14 µg ml^−1^ and FRAP value 5.87±0.08 mmol AAE g^−1^, while the lowest in F4 with EC_50/DPPH_ value 133.45±2.24 µg ml^−1^ and FRAP value 2.58±0.04 mmol AAE g^−1^. FRAP showed a similar trend to the results of DPPH assay. Although these two antioxidant assays with different mechanisms were used in this study, other antioxidant assays, such as peroxyl radical scavenging capacity or even assays for peroxidative/prooxidative enzymes should be included in further research, such as peroxyl radical scavenging capacity or even assays for peroxidative/prooxidative enzymes. Compared with the results from other plant materials, such as *Delonix regia* [18], *Litchi chinensis* [25] and *Garcinia mangostana* [32], proanthocyanidins from *C. tagal* leaves also exhibited substantial radical scavenging activity and reduction capacity.

**Figure 6:**
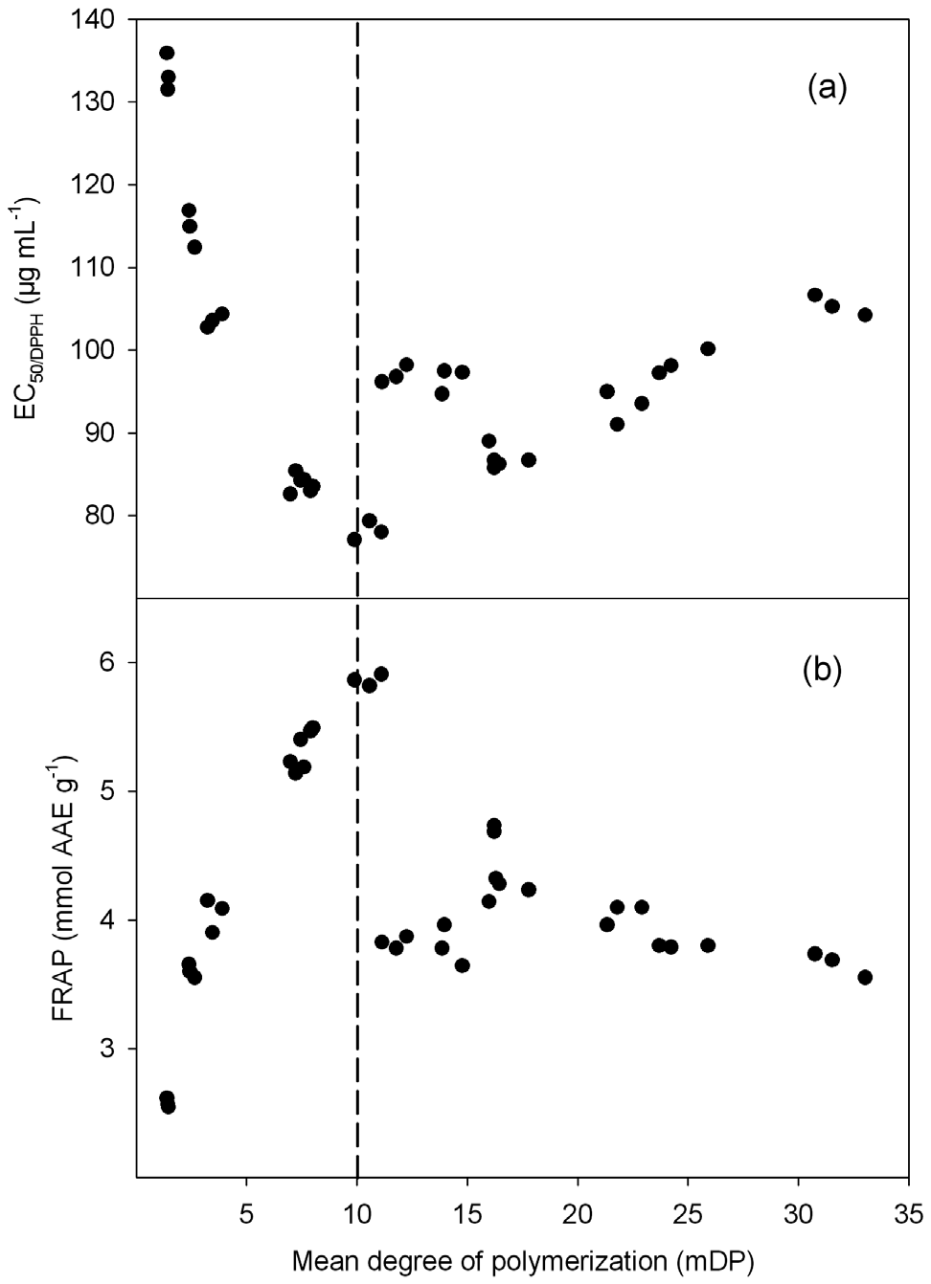
Regression analysis for mean degree of polymerization (mDP) and antioxidant activity. (a) EC_50/DPPH_ value and (b) FRAP value correlated with mDP for each proanthocyanidin subfraction from *C. tagal* leaves.

Regression analysis was performed to establish the relationship between antioxidant activities and mDP (Figure 6). It demonstrated that antioxidant activities was positively correlative with mDP when mDP <10, while dropped and then remained at a level with around 95 µg ml^−1^ for EC_50/DPPH_ value and 4 mmol AAE g^−1^ for FRAP value (similar to the antioxidant level of mDP = 5). Although some researchers attempted to study this relationship of proanthocyanidins, the work was limited to low oligomers and low DP. For instance, Gaulejac et al. [44] found an increase in the activity of procyanidins, but their work only focused on the oligomers from 1 to 4 units. Es-Safi et al. [24] simply compared the commercial standard substances (catechin and B_3_ procyanidin dimer) with pear juice polymeric proanthocyanidins. Jerez et al. [42] showed an increase of the antiradical activity of *Pinus pinaster* and *Pinus radiata* procyanidins up to 6–7 mDP (the mDP obtained by thiolysis with cysteamine). Recently, Zhou et al. [32] reported an increasing antioxidant activity of mangosteen tannins with mDP between 2.71 and 9.27, but decreasing in mDP = 16.80, and proposed that 9–10 mDP could be considered as a dividing and critical point for predicting the structure-activity of mangosteen condensed tannins, which was confirmed with the present results. However, all those previous works could not provide more information for the higher DP. In this study, a decrease and then remain at a lower antioxidant level was showed when mDP >10. However, the antioxidant activity based on the DPPH test in the present study could be influenced by the amount of total polyphenols. Zhou et al. [32] also showed linear relationships between DPPH and total polyphenol content. Similarly, antioxidant tests were positively correlated with total polyphenols [32], [45], [46], [47]. So only the mDP data may not be sufficient to explain the differences in EC_50_ and it is better to explain the differences in EC_50_ with reference to total phenolics and ECT in each fraction. Further investigations on the relationships between antioxidant activity and polyphenols in mangrove leaves and their mechanisms are required. Further investigations on the relationships between antioxidant activity and polyphenols in mangrove leaves and their mechanisms are required.

Mangrove tannins from *C. tagal* leaves were successfully characterized by ^13^C-NMR, MALDI-TOF MS, HPLC-ESI MS and column chromatographic fractionation. They had substantial DPPH free radical scavenging ability and ferric reducing antioxidant power, which could be used as a new source of antioxidants. The major challenge in the research on condensed tannins is probably the difficulty in obtaining them in an individual molecular form. The complete purification of a procyanidin with a DP above five is almost impossible. Therefore, for studying their structures and properties, more or less mixtures polymerized are often employed [48]. More, the synergistic effects of active mixtures make plant extracts and fractions more interesting than the pure compounds for functional food applications [42]. In the present study, an effective fractionation method was established to elucidate more about the relationships between DP and antioxidant activities. The established relationships could be used as a theoretical method for predicting the structure-activity of proanthocyanidins.
